# Analysis of the Relationships between Waste Cooking Oil Qualities and Rejuvenated Asphalt Properties

**DOI:** 10.3390/ma10050508

**Published:** 2017-05-06

**Authors:** Dong Zhang, Meizhu Chen, Shaopeng Wu, Jingxiang Liu, Serji Amirkhanian

**Affiliations:** State Key laboratory of Silicate Materials of Architectures, Wuhan University of Technology, Wuhan 430070, China; pytmac@whut.edu.cn (D.Z.); wusp@whut.edu.cn (S.W.); liujingx@whut.edu.cn (J.L.); serji.amirkhanian@gmail.com (S.A.)

**Keywords:** waste cooking oil, aged asphalt, rejuvenation, rheological properties, chemical composition, grey correlation analysis

## Abstract

Waste cooking oil (WCO), in many cases, can rejuvenate aged asphalt and restore its properties. However, the influence of WCO qualities on rejuvenation behaviors of aged asphalt has not been investigated in detail. The objective of this paper was to evaluate the effects of WCO viscosity and acid value on the basic, rheological, and chemical properties of a typical rejuvenated asphalt. Penetration, ring and ball (R and B) softening point, and ductility were tested to evaluate the influence of WCO qualities on basic properties of rejuvenated asphalts. Then, the rheological properties of rejuvenated asphalt were characterized based on rotational viscometer (RV), dynamic shear rheometer (DSR), and bending beam rheometer (BBR) test results. Further, SARA (saturates, aromatics, resins, and asphaltenes) fraction analysis and Fourier transform infrared spectroscopy (FTIR) tests were performed to investigate the effects of WCO qualities on asphalt chemical composition. Finally, grey correlation coefficients were calculated and the relationships between WCO qualities and rejuvenated asphalt properties were quantitatively evaluated. The experimental results indicated that WCO qualities influence the rejuvenation behaviors of aged asphalt significantly, and the WCO with higher qualities (low acid value and viscosity, as defined in this research) tends to achieve better rejuvenation effects. Based on the results of grey correlation analyses, the acid value is, relatively, a better indicator than viscosity in predicting the rejuvenation efficiency of WCO. The rejuvenation thresholds of WCO are varied with the categories of properties of rejuvenated asphalts, and WCO with an acid value of 0.4–0.7 mg KOH/g, or a viscosity of 140–540 mm^2^/s, can meet all of the performance requirements for asphalt rejuvenation used in this research.

## 1. Introduction

Asphalt binders have been widely utilized in pavement construction for their extraordinary elasticity, cohesion, adhesion, and stiffness. Currently, over 80% of expressways worldwide are constructed with hot-mix asphalt (HMA) [1,2]. However, rutting, stripping, cracks, subsidence, and potholes occur frequently during the life of the pavement, which results in the shortening of the road lifespan [3,4,5]. Therefore, a massive amount of reclaimed asphalt pavement (RAP) is collected every year due to road maintenance. 

RAP has been applied for many years in constructing new pavements because it contains valuable binders and aggregates but, in some cases, they have not been fully utilized [6,7]. In addition, the premature fatigue and low-temperature cracking failures of RAP have limited its extensive utilization in pavement [8,9,10]. To solve these problems, rejuvenators, which are usually comprised of low-viscosity components (asphaltenes and other additives) that could improve the performance of asphalt binders have been introduced to the mixtures containing RAP [11]. However, the large-scale application of rejuvenators has been limited for many reasons, including high cost of materials and high dosage in utilization, which will elevate the construction budget and difficulty of finishing the project within the budget [12]. In order to meet the high demand for asphalt recycling agents, waste cooking oil (WCO) was considered, researched, and applied in asphalt rejuvenation based on their similar chemical constituents to conventional rejuvenators. Asili and Zargar [13,14] verified the possibility of using WCO as an alternative rejuvenator for aged asphalt by comparing the physical, chemical, and rheological properties of aged asphalt and WCO-rejuvenated asphalt. Chen [15,16] investigated the optimum dosage of WCO for asphalt recycling, and founded that WCO can restore the high-temperature rheological properties of aged asphalt, but its low-temperature flexibility and elasticity need to be further improved. Su [17] investigated the possibility of using microcapsules containing WCO to rejuvenate aged asphalt. The results proved that the WCO can be encapsulated, and the synthetic WCO microcapsule can survive in melting asphalt, as well as recover its virgin properties. Yu [18] discussed the rejuvenation effects of waste vegetable oils on the rheological, microscopic, and chemical characterization of RAP binders. The results denoted that adding waste vegetable oils can restore the rheological properties and reproduce the surface microstructures of aged binders.

Notwithstanding WCO having distinct rejuvenation effects on aged asphalt binder, little information is available regarding the relationships between WCO properties and the effects on the asphalt rejuvenation. Therefore, the objective of this study is to investigate the effects of WCO quality on basic, chemical, and rheological properties of aged asphalt. A series of laboratory experiments, including penetration testing, ring and ball (R and B) softening point testing, ductility testing, dynamic shear rheometer (DSR) testing, bending beam rheometer (BBR) testing, SARA (saturates, aromatics, resins, and asphaltenes) fraction analysis, and Fourier transform infrared spectroscopy (FTIR) testing were conducted. In addition, the grey correlation analyses were performed to systematically investigate the influence of WCO qualities on properties of the rejuvenated asphalts.

## 2. Experiment Materials

### 2.1. Asphalt

One asphalt binder, AH-90 (KOCH Bitumen Co., Ltd., Wuhan, China) (referred to as A_0_), which is extensively utilized in China, was selected for this research. Its basic properties were tested and are presented in Table 1, accompanied by the basic properties of short-term and long-term aging asphalts.

### 2.2. Waste Cooking Oil (WCO)

Restaurant-recycled WCO was selected, in most research projects, to investigate the influence of WCO on asphalt performance. However the qualities of the WCO, such as viscosity, density, acid value, impurity content, and water content are uncontrollable and, thus, the experimental results are subject to uncertainty. Therefore, fresh soybean oil (referred to as W_0_) was selected and heated continuously to fabricate a controlled source of WCO in this paper. Wan et al. suggested that the acid value of WCO influences the performance of WCO-modified asphalt significantly, and the WCO with the lower acid value modifies the rheological properties better [19]. In addition, the American Society for Testing and Materials (ASTM) suggested that the viscosity at 60 °C is a critical indicator to classify hot-mix recycling agents. Therefore, the WCO qualities were characterized by the acid value and viscosity in this research project. 

The WCO samples were fabricated at a temperature of 270 °C and a stirring rate of 1200 rpm for 2 h, 4 h, 6 h, 8 h, 10 h, 12 h, 14 h, and 16 h, respectively. The obtained WCOs were referred to as W_1_–W_8_, respectively. The acid value and viscosity of WCO were tested in accordance with ASTM D1980 and ASTM D4552, respectively, and the results were demonstrated in Table 2. It can be seen that the acid values and viscosity values of WCO increased with the increase in heating time, distinctly. In most cases, a prolonged heating time meant a lower-quality WCO.

## 3. Research Methods

### 3.1. Preparation of Aged Asphalt Binder

Laboratory-accelerated aging was applied to prepare the aged asphalt binder in this research. Firstly, a rolling thin film oven test (RTFOT), conducted at 163 °C for 85 min, was selected to determine the approximate change in the properties of the asphalt during conventional hot-mixing (ASTM D2872). Secondly, a pressure aging vessel (PAV), simulating the in-service oxidative aging that emerge in asphalt binders during pavement service, was performed after RTFOT at 100 °C for 20 h with a pressure of 2.1 ± 0.1 MPa (ASTM D6521). In this research, the aged asphalt binder obtained from PAV after RTFOT was referred to as B_0_. Conventional properties of RTFOT-aged binder and PAV-aged binder are shown in Table 1. 

### 3.2. Preparation of the Rejuvenated Asphalt Binder

Rejuvenated asphalt binders were prepared by means of mixing laboratory accelerated-aging asphalt B_0_ with different WCOs (W_1_~W_8_). The blending process was conducted by a propeller mixer at a constant speed of 1200 rpm for 15 min, and the experimental temperatures were selected to be 130 °C. The proportion of WCO was 6.0 wt % of aged asphalt binder based on a previous study [15]. The rejuvenated asphalt binders were referred to as A_1_–A_8_, respectively.

### 3.3. Basic Properties Tests

To evaluate the influence of WCO quality on the basic properties of rejuvenated binders, penetration, ring and ball (R and B) softening point, and ductility were tested in this research. 

Penetration testing is a method to examine the consistency and deformation resistance of asphalt binders, as well as viscosity, under a certain condition, and it was implemented with a standard load of 100 g and a time of 5 s at 25 °C (ASTM D5). 

R and B softening point testing was applied to denote the temperature sensitivity of the rejuvenated asphalt. High temperature susceptibility is ideal when asphalt has a higher softening point value. The experiment was conducted at a constant heating rate of 5 °C/min (ASTM D36). 

Ductility testing was conducted at an elongation rate of 5 cm/min and a temperature of 5 °C in this research to evaluate the tensile deformation and flexibility of asphalt at low temperatures (ASTM D113). Asphalt binder with a lower ductility value is considered as having poor thermal cracking resistance in service.

### 3.4. Rheological Properties Tests

A rotational viscometer (RV), dynamic shear rheometer (DSR), and bending beam rheometer (BBR) were employed to investigate the rheological properties of rejuvenated binders in a wide temperature range. 

Viscosity is usually selected to characterize the shear resistance of asphalt binder under an external force. In this research, a Brookfield Model DV-III viscometer (THERMOSEL, BROOKFIELD, Stoughton, MA, USA) and Thermosel temperature control system (THERMOSEL, BROOKFIELD, Stoughton, MA, USA) were utilized and the rotational viscosity at 135 °C was tested to evaluate the mixing and compacting characteristics of HMA (ASTM D4402).

According to ASTM D7175, DSR (Anton Paar Physica MCR 301, Vienna, Austria) temperature sweep tests from 30 °C to 80 °C were performed to study the permanent deformation resistance of rejuvenated asphalts by means of constructing the rutting parameter (G*/sinδ) curves. DSR frequency sweep tests from −10 °C to 60 °C at 10 °C intervals with frequencies from 0.1 rad/s to 100 rad/s were conducted, and the time-temperature superposition principle (TTSP) was selected to construct master curves for complex modulus G* and phase angle *δ* at a reference temperature of 20 °C. The controlled strain mode with an applied strain of 0.5% was selected to ensure the tests remained in the linear visco-elastic region.

A Cannon TE-BBR was applied to conduct tests at −12 °C and −18 °C, in order to determine the cracking resistance of virgin, aged, and rejuvenated asphalt binders at low temperatures based on ASTM D6648.

### 3.5. Chemical Properties Tests

Thin-layer chromatography with flame ionization detection (TLC-FID, IATROSCAN MK-6, manufactured by Mitsubishi Kagaku Iatron, Inc., Tokyo, Japan) was employed to determine saturates, aromatics, resins, and asphaltene (SARA) fractions of rejuvenated asphalt binder. First, the asphalt was dissolved in dichloromethane with 2 wt % concentration. Then the chromarods were cleaned and activated in the FID-flame, and 1 μL of solution was spotted on the start of a chromarod using a micropipette. The separation was performed using a three-stage process. The first development was in n-heptane, the second stage was in toluene/n-heptane (80/20 by volume), and the last development was in dichloromethane/methanol (95/5 by volume). Saturates, aromatics, and resins were eluted stepwise, and the most polar asphaltenes remained at their original place on the chromarod. Finally, the chromarod was dried at 80 °C for 1 min in the FID, the individual separated zones were ionized in the hydrogen flame, and the ionized currents were recorded. The applied scan rate was 40 s/scan, and the air and hydrogen flows were 1500 mL/min and 160 mL/min, respectively. For each sample, five replicate tests were performed and the average value of the five readings was considered as the result.

Fourier transform infrared spectroscopy (FTIR, Nicolet™ 6700, Thermo Fisher Scientific Waltham, MA, USA) was applied to determine the functional characteristic of asphalt before and after rejuvenating. Specimens were prepared by dissolving the asphalt in carbon disulfide solvent with 5 wt % concentration. Then, the solutions were dropped into a blank KBr salt plate to fabricate a thin film. The carbon disulfide solvent was then evaporated and the finished specimens were placed into the infrared spectrometer for testing. The scan ranged from 4000 cm^−1^ to 500 cm^−1^ with a 4 cm^−1^ resolution.

### 3.6. Grey Correlation Analysis

Grey correlation analysis (GCA) is a mathematical analysis method to deal with finite and seemingly irregular data, thus determining whether a relationship among these data is close [20]. The principle of GCA is generally based on the similarity degree judgment among geometric shapes of array curves, and a closer curve indicates a greater correlation among the relative data arrays [21]. The grey correlation coefficient can be also utilized to provide the quantitative expression of GCA, and a greater correlation coefficient means a higher similarity among arrays. Previous researchers have indicated that GCA is applicable to study the performance of an asphalt and an asphalt mixture, and the analyses results are accurate and reliable [22,23]. 

The detailed calculating methods of grey correlation coefficient are as follows:

Supposing there is a series of data arrays:

{X_0_^(0)^(r)} r = 1, 2, 3, ..., N_0_
{X_1_^(0)^(r)}, r = 1, 2, 3, ..., N_1_
{X_2_^(0)^(r)}, r = 1, 2, 3, ..., N_2_
... ...
{X_k_^(0)^(r)}, r = 1, 2, 3, ..., N_k_

where *N_1_, N_2_, ..., N_k_* are all the natural numbers and they might not be equal. The *k* arrays express *k* factors. Array *{X_0_^(0)^(r)}* is named the main-array and arrays *{X_m_^(0)^(r)} (m = 1, 2, 3, ..., k)* are named sub-arrays. The main-array and sub-arrays must be transformed to be dimensionless to make sure the consistency of the analysis results. The methodologies can be utilized for normalizing in the grey system, including the initial value processing method, maximum/minimum method, and equalization method, etc. This paper adopts the equalization method, which divides the data in the original arrays by their average, to process the data.

The normalization methods are as follow:

(1) $$\left\{Y_{m}\left(r\right)\right\}=\left\{X_{m}^{\left(0\right)}\left(r\right)\right\}/{\bar{X}}_{m}$$

(2) $${\bar{X}}_{m}=\frac{1}{N_{m}}\left\{\overset{N_{m}}{\underset{r=1}{\sum }}X_{m}^{\left(0\right)}\left(r\right)\right\}$$

where *{Y_m_(r)}* is the normalized array, *X_m_* is the average value of sub-arrays *{X_m_^(0)^(r)}*, *r = 1, 2, 3, ..., N_m_ , m = 0, 1, 2, 3....*

The correlation coefficient between main-array and sub-arrays can be described as:

(3) $$\gamma_{i}=\frac{1}{N}\overset{N}{\underset{k=1}{\sum }}\xi_{i}\left(k\right)$$

In which,

(4) $$\xi_{i}\left(k\right)=\frac{\underset{i}{min}\;\underset{k}{min}\left|Y_{m}\left(k\right)-Y_{0}\left(k\right)\right|+\rho \;\underset{i}{max}\;\underset{k}{max}\left|Y_{m}\left(k\right)-Y_{0}\left(k\right)\right|}{\left|Y_{m}\left(k\right)-Y_{0}\left(k\right)\right|+\rho \;\underset{i}{max}\;\underset{k}{max}\left|Y_{m}\left(k\right)-Y_{0}\left(k\right)\right|}$$

where *ρ* is distinguishing coefficient, *ρ*∈(0, 1). *ρ* is normally defined as 0.5.

## 4. Results and Discussion

### 4.1. Basic Properties

**Figure 1:**
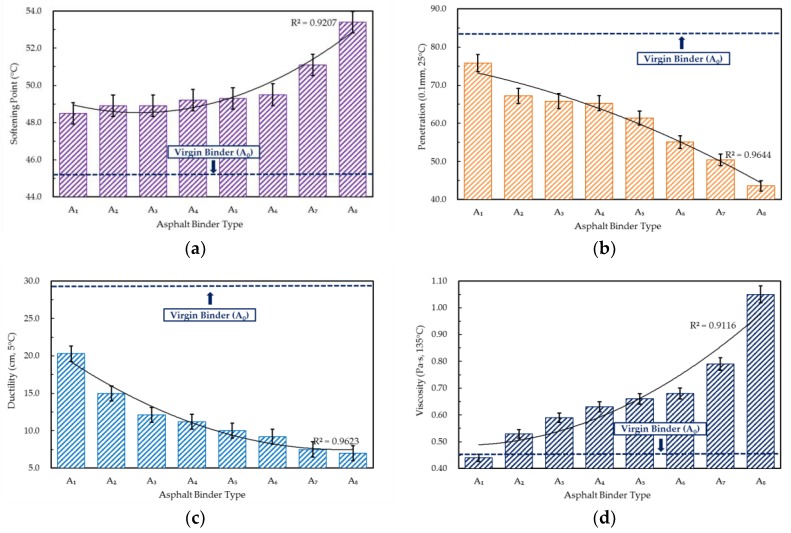
Basic properties of virgin, aged, and rejuvenated asphalts. (**a**) Softening point of virgin, aged, and WCO-rejuvenated asphalts; (**b**) Penetration of virgin, aged, and WCO-rejuvenated asphalts; (**c**) Ductility of virgin, aged, and WCO-rejuvenated asphalts; (**d**) Rotational viscosity of virgin, aged, and WCO-rejuvenated asphalts.

The analyses of the basic properties of rejuvenated asphalt, including softening point, penetration, and ductility are demonstrated in Figure 1a–c, respectively. It can be observed that with the deterioration of the WCO quality, the penetration and ductility of rejuvenated asphalt decreased rapidly while the softening point increased slowly. These results indicated that the WCO with superior qualities was more effective on the restoration of the consistency, flexibility, and temperature susceptibility of an aged asphalt. However, despite the basic properties have been improved due to the addition of WCO, the performance gaps between WCO-rejuvenated asphalts, and the virgin asphalt (A_0_) still existed, which meant the rejuvenation effects of WCO was limited. 

The current penetration grading system is primarily based on empirical methods for analysis and testing, which means that these basic properties of asphalt (e.g., penetration, ductility, softening point) cannot precisely reveal its pavement performance. Different asphalts which have the same penetration grade, even the identical penetration value, may present different properties in a practical application. Therefore, in order to investigate the rejuvenation effects of WCO accurately, rheological and chemical properties tests were subsequently conducted.

### 4.2. Rheological Properties 

#### 4.2.1. Rotational Viscosity

Rotational viscosity test results of asphalts rejuvenated by different WCOs are illustrated in Figure 1d. According to the results demonstrated above, the viscosity values of the rejuvenated asphalt increased gradually with the WCO quality degraded, and the rejuvenated asphalt A_8_ had an almost identical viscosity value compared to the aged asphalt B_0._ This result indicated that the selected WCO which had better qualities than that of W_8_ (acid value is 3.231 mg KOH/g, viscosity is 1143.46 mm^2^/s) can improve the fluidity of aged asphalt, as well as the operation in the field (e.g., compaction) and energy consumption of the system HMA. On the other hand, it was notable that the viscosity value of virgin asphalt A_0_ was between that of the rejuvenated asphalts A_1_ and A_2_, which meant the WCO with and acid value of 0.381 mg KOH/g or viscosity of 144.27 mm^2^/s can restore the viscosity of aged asphalt to that of the virgin asphalt. Therefore, it can be concluded that using WCO with acid values ranging from 0.4 to 3.2 mg KOH/g or viscosity values ranging from 140 to 1140 mm^2^/s can achieve the restoration of the aged asphalt viscosity in this case. Normally, WCO with lower acid value or viscosity can also be selected for asphalt rejuvenation, and it may obtain an even lower viscosity value than the virgin asphalt. However, an excessively low viscosity of an asphalt would lead to adhesion reduction and performance damage of an asphalt mixture. Consequently, it was advisable to keep the acid value and viscosity of WCO in a reasonable range.

#### 4.2.2. Rutting Parameter

**Figure 2:**
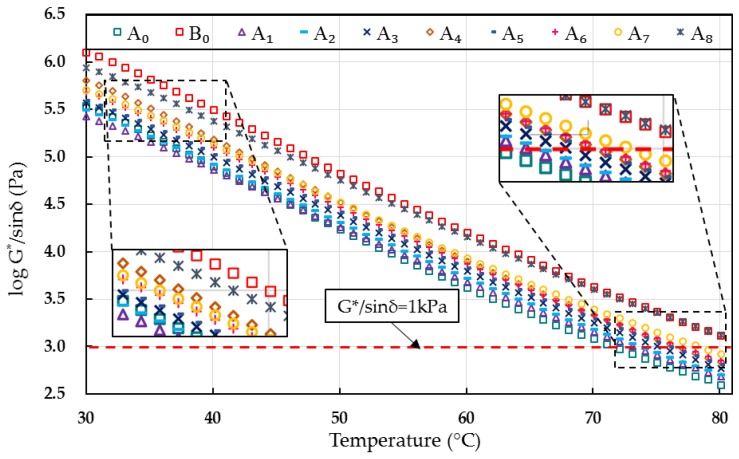
Rutting parameter of virgin, aged, and rejuvenated asphalts.

Rutting parameter (G*/sinδ) reflects the unrecoverable deformation of asphalts during the loading process. Asphalt binders with a higher value of G*/sinδ, but a lower flow deformation at high temperatures, is desirable [16]. Figure 2 exhibits the influence of WCO quality on G*/sinδ of rejuvenated binders.

It can be observed that the G*/sinδ values of rejuvenated binders strongly depended on the qualities of WCO, as well as the test temperatures. Generally, the rutting parameters of rejuvenated asphalts decreased as the test temperatures and WCO quality increased. Specifically, the G*/sinδ values of rejuvenated asphalt binders containing different WCO were lower than that of the aged asphalt B_0_, but higher than that of the virgin asphalt A_0_. However, as the figure illustrated, the rejuvenated asphalts A_1_ and A_2_ exhibited lower G*/sinδ values than the virgin asphalt at the temperatures ranging from 30 °C to 40 °C, while the G*/sinδ of rejuvenated asphalt A_8_ was higher than that of the aged asphalt at the temperatures ranging from 70 °C to 80 °C_._ Therefore, WCOs with acid values ranging from 0.4 to 3.2 mg KOH/g or viscosity values ranging from 210 to 1140 mm^2^/s were recommended in this research to restore the high-temperature properties of the aged asphalt.

#### 4.2.3. Critical High Temperature

**Figure 3:**
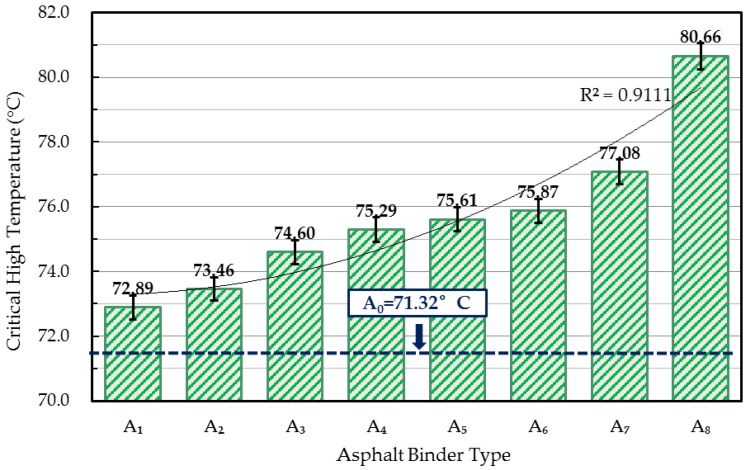
Critical high temperature of rejuvenated asphalts.

Critical high temperature is a threshold that the time-temperature superposition principle (TTSP) is no longer applicable when the mixture temperature exceeds that value [24]. Figure 3 demonstrates the influence of WCO qualities on the critical high temperature of rejuvenated asphalts. It was noticeable that these values of rejuvenated asphalt binders were strongly dependent on the qualities of WCO. With the WCO qualities increasing, the critical high temperature values decreased, and the value of rejuvenated asphalt A_1_ (with a failure temperature of 72.89 °C) was scarcely higher than that of the virgin asphalt (with a critical high temperature of 71.32 °C). These findings were consistent with the rutting parameter analyses that WCOs with lower acid value or viscosity were desirable for the high-temperature property recovery of aged asphalt.

#### 4.2.4. Complex Modulus and Phase Angle Master Curves

To fully characterize the rheological properties of the asphalt, TTSP was applied to relate the equivalency between temperature and frequency and thereby produced the master curves of the complex modulus and phase angle. 

The selected reference temperature *T_r_* was 20 °C in this research, and the shift factor *α(T)* was determined by the Williams, Landel, and Ferry (WLF) model using the following equation [25]:

(5) $$log\alpha \left(T\right)=-\frac{C_{1}\left(T-T_{r}\right)}{C_{2}+\left(T-T_{r}\right)}$$

where *T* was the actual loading temperature, *C_1_* and *C_2_* were empirically-determined coefficients. The extended frequency scale operated in the master curve was referred to as the reduced frequency and it was defined as:

(6) $$\log f_{r}=logf+log\alpha \left(T\right)$$

where *f_r_* was the reduced frequency, *f* was the actual loading frequency. The master curves were modeled using the Christensen, Anderson, and Marasteanu (CAM) model [26], and the complex modulus was defined as below:

(7) $$G^{*}=G_{g}\times {\left[1+{\left(\frac{f_{c}}{f_{r}}\right)}^{\vartheta }\right]}^{-\frac{\omega }{\vartheta }}$$

The phase angle *δ* was defined as:

(8) $$\delta =\frac{90\omega }{\left[1+{\left(\frac{f_{c}}{f_{r}}\right)}^{\vartheta }\right]}$$

where G* is the complex modulus, *δ* is the phase angle, *G_g_* is the glassy modulus, *f_c_* is the crossover frequency, and *ω* is the shape-fitting parameter.

**Figure 4:**
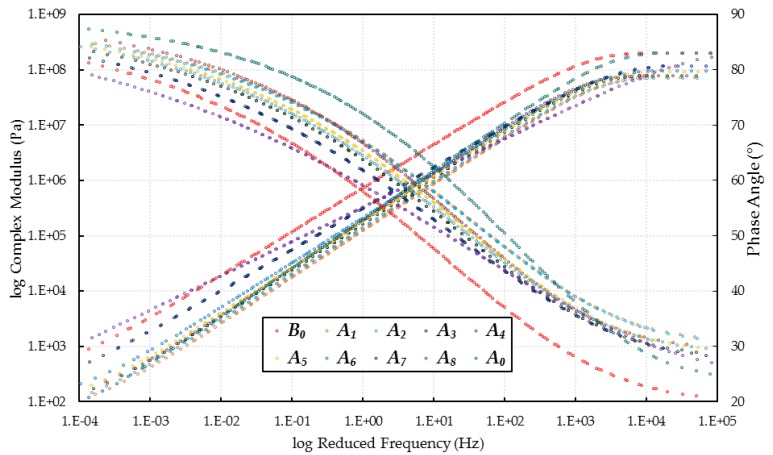
G* and *δ* master curves of virgin, aged, and WCO-rejuvenated asphalts.

Complex modulus G* and phase angle *δ* master curves of virgin, aged, and WCO-rejuvenated asphalts at a reference temperature of 20 °C are shown in Figure 4. It can be noticed that at low frequencies (high temperatures), the G* values of WCO-rejuvenated asphalts were lower than that of aged asphalt (A_0_), while the *δ* values were higher than that of A_0_, except for A_8_. These results indicated that the addition of WCO had a great effect on the permanent deformation resistance restoration of an asphalt, and the WCO with acid values exceeding 3.2 mg KOH/g, or with viscosities higher than 1140 mm^2^/s, were inapplicable to restore the high-temperature properties. Moreover, the high-temperature rheological properties of rejuvenated asphalts were varied with different WCOs. With the advancing of WCO qualities, the complex modulus and phase angle master curves of rejuvenated asphalts were approaching that of the virgin asphalt, which meant the rejuvenation effects were improving. Therefore, it can be concluded that the WCOs with acid values lower than 3.2 mg KOH/g, or with viscosities lower than 1140 mm^2^/s, were suitable for restoring the high-temperature rheological properties of aged asphalt, and the recovery efficiency increased with the increase in WCO’s qualities.

As for the rheological properties at high frequencies (low temperatures), the rejuvenated asphalts exhibited higher *δ* values and lower G* values than virgin asphalt, which indicated that adding WCO was effective for restoring the low-temperature cracking resistance of an asphalt. Furthermore, the WCO with lower acid values and viscosities seemingly had greater performance improvement for the rejuvenated asphalt. Therefore, the low-temperature rheological properties of WCO-rejuvenated asphalt were further investigated by BBR tests.

#### 4.2.5. Stiffness and *m*-Value

**Figure 5:**
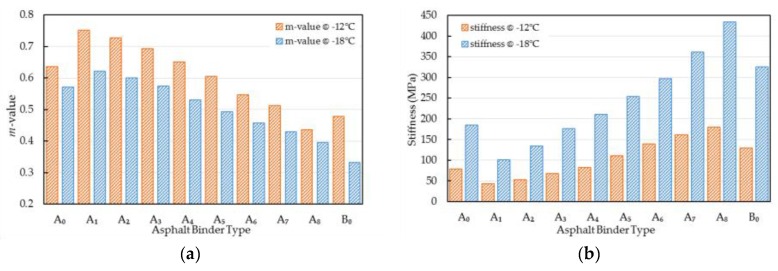
*m*-value and stiffness of virgin, aged, and WCO-rejuvenated asphalts. (**a**) m-value of virgin, aged, and WCO-rejuvenated asphalts at −12 °C and −18 °C; (**b**) Stiffness of virgin, aged, and WCO-rejuvenated asphalts at −12 °C and −18 °C.

Two parameters can be determined in the BBR tests, the creep stiffness is a measure of an asphalt resisting the constant loading, and the creep rate (also called *m*-value) is used to characterize the stiffness changing rule of an asphalt as the loads are applied. Figure 5 illustrated the stiffness and *m*-value of virgin, aged, and WCO-rejuvenated asphalts. It can be noticed that the stiffness increased and the *m*-value decreased for all asphalt binders as the experimental temperature decreased (−12 °C to −18 °C, in this case), which meant the temperature reduction, as expected, would increase the risk of thermal cracking. In addition, the restorative effects for thermal cracking resistance varied with the qualities of WCO. The stiffness values increased while the *m*-value decreased with the decreasing WCO qualities, and the stiffness and *m*-value of virgin asphalt were similar to that of the rejuvenated asphalt A_4_. Therefore, the conclusion can be drawn that the restorative effects of thermal cracking resistance decreased with the qualities of the applied WCO, and the thresholds for WCO used in the rejuvenation of aged asphalt were 0.7 mg KOH/g for the acid value and 420 mm^2^/s for the viscosity. 

#### 4.2.6. Critical Low Temperature

**Figure 6:**
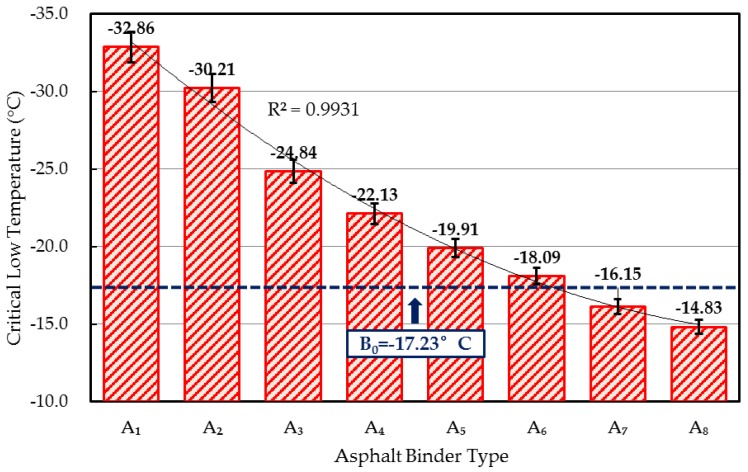
Critical low temperature of rejuvenated asphalts.

The critical low temperature of WCO-rejuvenated asphalts was determined from the BBR results. As illustrated in Figure 6, the critical low temperatures of rejuvenated asphalt binders were strongly influenced by the qualities of the WCO. Generally, with the WCO qualities decreased, the critical low temperatures were increased, and the value of rejuvenated asphalt A_7_ (critical low temperature was −16.15 °C) was barely higher than that of the aged asphalt (critical low temperature was −17.23 °C). These results were consistent with the thermal cracking resistance analyses that WCOs with lower acid value or viscosity were desirable for the low-temperature property recovery of aged asphalt.

### 4.3. Chemical Properties

#### 4.3.1. SARA Fractions

Asphalt is conventionally considered as a colloidal system which consists of high molecular weight asphaltene micelles and a low molecular weight oily medium (maltenes). The asphaltene molecule acts as the colloidal nucleus, while the aromatic resins enclose that to form an asphaltene micelle. If there are sufficient quantities of resins and aromatics, the asphaltenes can be fully peptized and result in an effective mobility of micelles within the asphalt. These are regarded as “sol” type asphalts, otherwise it is called as “gel” type asphalt. The degree of asphaltenes peptizing has a great influence on the resultant viscosity of the system. Gaestel has defined a colloidal instability index (I_C_) as a ratio of the sum of the amounts of asphaltenes and saturates to the sum of the amounts of aromatics and resins, in order to determine the colloidal structure of asphalts [27].

Table 3 exhibited the SARA (saturates, aromatics, resins, and asphaltenes) fractions of virgin, aged, and WCO-rejuvenated asphalts. Compared with the constituents of virgin asphalt (A_0_), aged asphalt (B_0_) exhibited more resins and asphaltenes, but fewer saturates and aromatics. The reason might be that the naphthene aromatics were converted in part to polar aromatics, which later transformed into asphaltenes [28]. The trends of weight percent of all four fractions in rejuvenated asphalt were also distinct. The results demonstrated an increase in the asphaltene and resin fractions and a decrease in aromatic and saturate fractions with the WCO qualities decreasing, and these phenomenon can be explained by the dilution of resins and asphaltenes arose from the varied qualities of WCOs.

Table 3 shows that the virgin asphalt has an I_C_ value of 0.3048, while the value increased to 0.4116 after aging, which indicated that a colloidal change happened due to the sequential transformation of aromatics to resins to asphaltenes. As for the I_C_ values of rejuvenated asphalts, a significant decrease can be noticed compared with the aged asphalt, which meant the colloidal systems were transformed from gel-type to sol-type. Specifically, the I_C_ values of rejuvenated asphalts A_1_, A_2_, A_3_ and A_4_ (I_C_ = 0.2943, 0.2999, 0.3135, and 0.3127, respectively) were similar to that of the virgin asphalt (I_C_ = 0.3048), while the corresponding values of the rejuvenated asphalts A_5_, A_6_, A_7_, and A_8_ (I_C_ = 0.3473, 0.3607, 0.3349, and 0.3452, respectively) were higher than that of the virgin asphalt. Consequently, the qualities of W_4_ (acid value of 0.727 mg KOH/g and viscosity of 420.20 mm^2^/s) were considered to be the thresholds for recovering the colloidal system of the rejuvenated asphalt.

#### 4.3.2. FTIR Spectra and Functional Group Index

Carbonyl (C=O) and sulfoxide (S=O) peak area intensities can reflect the aging and rejuvenating degree of an asphalt [29]. The functional group index can be calculated by the area of their bands by the following equations:

(9) $$I_{C=O}=\frac{Carbonyl\;peak\;area\;\left(centered\;around\;1700\;cm^{-1}\right)}{Peak\;area\;\left(\sum^{\text{​}}2000\;and\;600\;cm^{-1}\right)}$$

(10) $$I_{S=O}=\frac{Sulfoxide\;peak\;area\;\left(centered\;around\;1030\;cm^{-1}\right)}{Peak\;area\;\left(\sum^{\text{​}}2000\;and\;600\;cm^{-1}\right)}$$

where I_C=O_ is the C=O peak area intensity, and I_S=O_ is the S=O peak area intensity.

**Figure 7:**
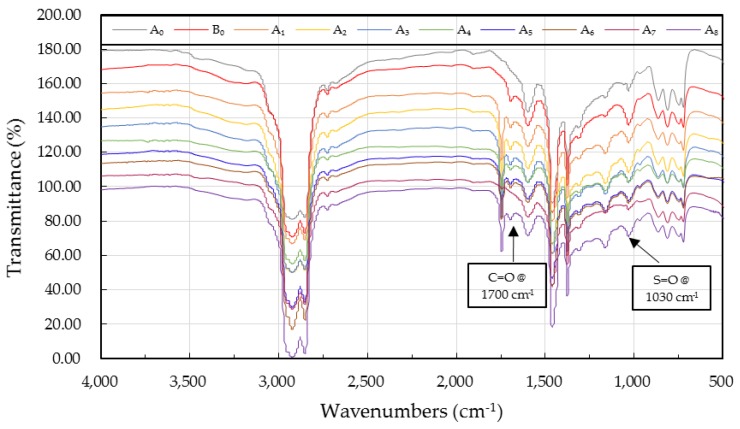
FTIR spectra of the virgin, aged, and the WCO-rejuvenated asphalt binders.

Figure 7 illustrates the FTIR spectra of virgin, aged, and the WCO-rejuvenated asphalt binders at the wavenumbers between 4000 cm^−1^ and 500 cm^−1^. Compared with the virgin asphalt binder (A_0_), aged asphalt (B_0_) exhibited a notable increasing in C=O and S=O peak areas. As for the rejuvenated asphalts, the C=O and S=O peak areas increased gradually with the applied WCO qualities decreasing, which meant the qualities of WCO had great influence on the chemical constituents of the rejuvenated asphalt, and the WCOs with lower acid values and viscosities could achieve better chemical performance. Furthermore, within the spectra of rejuvenated asphalt binders emerged a new absorption peak at 1746 cm^−1^, belonging to ester carbonyl functional group. The new absorption peak is the characteristic peak of soybean oil.

Quantitative analyses of functional group indices were performed and presented in Table 4. It can be observed that the I_C=O_ of virgin asphalt A_0_ was zero while the corresponding value of aged asphalt B_0_ (I_C=O_=0.031) was noticeably higher, which was consistent with the previous study of Wu et al. that the carbonyl index is available for evaluating whether it is virgin asphalt [30]. As for the rejuvenated asphalts, the I_C=O_ and I_S=O_ values increased gradually with the deterioration of the WCO qualities, which were in accordance with the results of FTIR spectra analyses in Figure 7. In general, the I_C=O_ values of rejuvenated asphalts were lower than that of aged asphalt (I_C=O_ = 0.031), except for rejuvenated asphalts A_6_ (I_C=O_ = 0.035), A_7_ (I_C=O_ = 0.040), and A_8_ (I_C=O_ = 0.053). The same trend can be noticed in the sulfoxide indices of rejuvenated asphalts. Consequently, the conclusions can be drawn from the FTIR spectra analyses and functional group index results that the recovery efficiency of rejuvenated asphalt chemical constituents decreased with the decreasing in qualities of the WCO. In addition, it can be concluded that the WCOs whose acid values were higher than 1.2 mg KOH/g or viscosities higher than 540 mm^2^/s were unsuitable for restoring the chemical composition of aged asphalt.

### 4.4. Grey Correlation Analysis

To investigate the relationship between WCO qualities and the rejuvenated asphalt properties more systematically, grey correlation analysis was conducted and the grey correlation coefficient was calculated to obtain the quantitative expressions. Based on the data listed in Table 5, the properties of rejuvenated asphalts, including penetration, softening point, ductility, viscosity, critical high temperature, carbonyl index, sulfoxide index, creep stiffness at −12 °C and −18 °C, *m*-value at −12 °C and −18 °C, and critical low temperature were regarded as main-arrays, and the acid value and viscosity of the WCO were considered as the sub-arrays. The results of the grey correlation coefficient are also listed in Table 5.

The results indicated the grey correlation coefficients of the WCO acid value and WCO viscosity were higher than 0.6000, in general, which meant that both of the two quality parameters of WCO had close relationships with the properties of the rejuvenated asphalt. In addition, the WCO acid value was the major influential factor compared with its viscosity, and these results indicate that the acid value of WCO had better predictive validity in rejuvenated asphalt properties than the WCO viscosity. 

Another notable consequence was that the grey correlation coefficients of the sulfoxide index and creep stiffness (ranging from 0.7000 to 0.8000) were significantly higher than that of other properties, which meant that the aging degree and thermal cracking resistance of rejuvenated asphalt can be predicted more precisely based on the acid value and viscosity of the applied WCO. 

## 5. Conclusions and Future Work

Based on the experimental results from an asphalt binder rejuvenated with different quality WCOs, in terms of a series of basic, chemical, and rheological properties tests, the following conclusions can be drawn:

1. WCO qualities influence the properties of the rejuvenated asphalt significantly, and the WCO with higher qualities (lower acid value and viscosity in this research) achieves superior rejuvenation effects of the aged asphalt.
2. Grey correlation analyses indicate that both WCO acid value and WCO viscosity have a close relationship with the properties of the rejuvenated asphalt, especially for the aging degree and thermal cracking resistance. The WCO acid value is a better criterion than WCO viscosity to predict the performance of the WCO-rejuvenated asphalt.
3. The rejuvenation thresholds of WCOs are varied with the property categories of the rejuvenated asphalts. For the high-temperature rheological properties, WCOs with acid values of 0.4–3.2 mg KOH/g or viscosities of 140–1140 mm^2^/s are preferable. For the low-temperature rheological properties and colloidal system stability, WCOs with acid values lower than 0.7 mg KOH/g or viscosities lower than 420 mm^2^/s are recommended. As for the chemical constituent recovery of an aged asphalt, WCO with acid values lower than 1.2 mg KOH/g or with viscosities lower than 540 mm^2^/s can achieve satisfactory results.
4. Comprehensive results show that WCOs with acid and viscosity values in the ranges of 0.4–0.7 mg KOH/g and 140–540 mm^2^/s, respectively, can meet all of the requirements for asphalt rejuvenation based on the materials used in this research.

Despite the effects of WCO qualities on the properties of rejuvenated asphalt was studied, much work should be conducted to further investigate the microstructure of the rejuvenated asphalt by adding WCO with different qualities. In addition, the influence of WCO qualities on reclaimed asphalt mixture performance and the energy efficiency of asphalt pavements in its life cycle need to be further researched. Finally, more quality indicators of WCO, such as relative density, water content, polycyclic aromatic hydrocarbons content, and peroxide value, can be taken into account to investigate their effects on rejuvenated asphalt in the future.

## Figures and Tables

**Table 1:** Basic properties of the asphalt binder utilized in this research project.

Asphalt Categories	Softening Point (°C)	Penetration (0.1 mm, 25 °C)	Ductility (cm, 15 °C)	Viscosity (Pa·s, 135 °C)
**AH-90 (A_0_)**	45.1	83.7	>100	0.45
**AH-90 RTFOT**	49.9	53.4	129	0.57
**AH-90 RTFOT+ PAV (B_0_)**	58.5	32.7	5	0.99

**Table 2:** Qualities of different WCOs.

Sample	W_0_	W_1_	W_2_	W_3_	W_4_	W_5_	W_6_	W_7_	W_8_
Heating time (h)	0	2	4	6	8	10	12	14	16
Acid value (mg KOH/g)	0.183	0.381	0.455	0.553	0.727	1.214	1.333	2.318	3.231
Viscosity (mm^2^/s)	55.31	144.27	208.56	300.22	420.20	540.95	693.05	866.10	1143.46
Density (kg/L)	0.904	0.908	0.911	0.916	0.921	0.928	0.935	0.941	0.955

**Table 3:** SARA fractions of virgin, aged, and rejuvenated asphalts.

Sample	Saturates	Aromatics	Resins	Asphaltenes	*I_C_*
A_0_	13.23	41.84	34.80	10.13	0.3048
B_0_	9.58	33.44	37.40	19.58	0.4116
A_1_	13.71	45.23	32.03	9.03	0.2943
A_2_	13.49	43.21	33.72	9.58	0.2999
A_3_	13.03	42.89	33.24	10.84	0.3135
A_4_	12.81	41.41	34.77	11.01	0.3127
A_5_	12.44	40.02	34.20	13.34	0.3473
A_6_	11.97	39.08	34.41	14.54	0.3607
A_7_	10.06	38.68	36.23	15.03	0.3349
A_8_	9.84	37.46	36.88	15.82	0.3452

**Table 4:** Functional group index of the virgin, aged, and rejuvenated asphalt.

Asphalt	A_0_	B_0_	A_1_	A_2_	A_3_	A_4_	A_5_	A_6_	A_7_	A_8_
Carbonyl index, I_C=O_	0.000	0.031	0.008	0.013	0.018	0.023	0.030	0.035	0.040	0.053
Sulfoxide index, I_S=O_	0.079	0.118	0.037	0.051	0.062	0.070	0.074	0.081	0.089	0.074

**Table 5:** Rejuvenated asphalt properties and the affecting factors.

Properties Categories	A_1_	A_2_	A_3_	A_4_	A_5_	A_6_	A_7_	A_8_	Factor Analysis	
									Acid Value (mg KOH/g)	Viscosity (60 °C, mm^2^/s)
Penetration (25 °C, 0.1 mm)	75.8	67.2	65.8	65.3	61.4	55.1	50.4	43.6	0.6513	0.5868
Softening point (°C)	48.5	48.9	48.9	49.2	49.3	49.5	51.1	53.4	0.6528	0.5883
Ductility (15 °C, cm)	20.3	15.0	12.1	11.2	10.0	9.2	7.5	7.0	0.6415	0.6522
Viscosity (135 °C, Pa·s)	0.44	0.53	0.59	0.63	0.66	0.68	0.79	1.05	0.6429	0.5465
Critical high temperature (°C)	72.89	73.46	74.60	75.29	75.61	75.87	77.08	80.66	0.6564	0.5808
Carbonyl index	0.008	0.013	0.018	0.023	0.030	0.035	0.040	0.053	0.6210	0.6424
Sulfoxide index	0.037	0.051	0.062	0.070	0.074	0.081	0.089	0.074	0.7777	0.7439
Stiffness @−12 °C (MPa)	43.1	53.2	67.4	82.3	110.5	138.8	161.2	179.6	0.8088	0.7245
Stiffness @−18 °C (MPa)	101.0	134.5	176.1	211.2	254.2	297.5	361.8	434.6	0.7532	0.6929
*m*-value @−12 °C	0.752	0.728	0.694	0.651	0.606	0.547	0.512	0.436	0.6314	0.5887
*m*-value @−18 °C	0.622	0.601	0.574	0.530	0.494	0.458	0.430	0.396	0.6173	0.5992
Critical low temperature (°C)	−32.86	−30.21	−24.84	−22.13	−19.91	−18.09	−16.15	−14.83	0.6300	0.6363
